# Immune Dysregulation in SARS-CoV-2 patients coinfected with *Mycobacterium tuberculosis* (*Mtb*) or HIV in China

**DOI:** 10.1186/s12889-024-17905-3

**Published:** 2024-02-22

**Authors:** Lei Li, Jianxiang Zhang, Ranran Sun, Hong Liu, Genyang Cheng, Feifei Fan, Chong Wang, Ang Li, Hongxia Liang, Zujiang Yu, Guiqiang Wang, Zhigang Ren

**Affiliations:** 1 Department of Infectious Diseases, The First Affiliated Hospital of Zhengzhou University, #1 Jianshe East Road, 450052 Zhengzhou, China; 2 State Key Laboratory of Antiviral Drugs, The First Affiliated Hospital of Zhengzhou University, 450052 Zhengzhou, China; 3 Department of Respiratory and Critical Care Medicine, The First Affiliated Hospital of Zhengzhou University, 450052 Zhengzhou, China; 4 Department of Nephrology, The First Affiliated Hospital of Zhengzhou University, 450052 Zhengzhou, China; 5 Department of Hematology, The First Affiliated Hospital of Zhengzhou University, 450052 Zhengzhou, China; 6 Precision Medicine Center, Gene Hospital of Henan Province, the First Affiliated Hospital of Zhengzhou University, 450052 Zhengzhou, China; 7 Department of Infectious Diseases and the Center for Liver Diseases, Peking University First Hospital, No. 8 Xishiku Street, Xicheng District, 100034 Beijing, China

**Keywords:** SARS-CoV-2, Delta variant, *Mycobacterium tuberculosis* (*Mtb*), HIV, Omicron variant

## Abstract

**Background:**

SARS-CoV-2 infections usually cause immune dysregulation in the human body. Studies of immunological changes resulting from coinfections with *Mycobacterium tuberculosis* (*Mtb*) or HIV are limited.

**Methods:**

We conducted a retrospective study focusing on patients with COVID-19. A total of 550 patients infected with SARS-CoV-2 were enrolled in our study and categorized into four groups based on the presence of coinfections; 166 Delta-infected patients, among whom 103 patients had no coinfections, 52 who were coinfected with *Mtb*, 11 who were coinfected with HIV, and 384 Omicron-infected patients. By collecting data on epidemiologic information, laboratory findings, treatments, and clinical outcomes, we analyzed and compared clinical and immunological characteristics.

**Results:**

Compared with those in the Delta group, the median white blood cell, CD4 + T-cell and B-cell counts were lower in the *Mtb* group and the HIV group. Except for those in the Omicron group, more than half of the patients in the three groups had abnormal chest CT findings. Among the three groups, there were no significant differences in any of the cytokines. Compared with those in the Delta group, the disease duration and LOS were longer in the *Mtb* group and the HIV group. For unvaccinated Delta-infected patients, in the *Mtb* and HIV groups, the number of B cells and CD4 + T cells was lower than that in the Delta group, with no significant difference in the LOS or disease duration. In the *Mtb* group, three (6%) patients presented with a disease duration greater than four months and had decreased lymphocyte and IL17A counts, possibly due to double infections in the lungs caused by SARS-CoV-2 and *M. tuberculosis.*

**Conclusions:**

We found that SARS-CoV-2 patients coinfected with *Mtb* or HIV exhibited a longer disease duration and longer LOS, with a decrease in B cells and CD4 + T cells, suggesting that these cells are related to immune function. Changes in cytokine levels suggest that coinfection with *Mtb* or HIV does not result in dysregulation of the immune response. Importantly, we discovered a chronic course of coinfection involving more than four months of *Mtb* and SARS-CoV-2 infection.

**Supplementary Information:**

The online version contains supplementary material available at 10.1186/s12889-024-17905-3.

## Introduction

Since 2020, coronavirus disease 2019 (COVID-19) has spread worldwide, causing a severe public health care system crisis. To date, there are five main lineages of variants, namely Alpha, Beta, Gamma, Delta and Omicron [1]. The Delta variant with strong receptor binding and immune escape ability is considered among the most contagious variants known thus far; these variants have continued to evolve and mutate [1–4]. Moreover, it was reported that the Delta variant has characteristics of faster viral replication, higher viral load and greater infectiousness [5], and infection with Omicron is associated with significantly reduced severity and mortality compared with COVID-19 caused by previous variants [6, 7]. A study suggested that the current generation of vaccines will provide protection against severe acute respiratory syndrome coronavirus 2 (SARS-CoV-2), although reduced titers may lead to breakthrough infections [4, 8]. As of 16 August 2023, more than 769 million people worldwide were confirmed to be infected with SARS-CoV-2 [9]. SARS-CoV-2 infection has been shown to have more adverse outcomes in people with underlying disease. Underlying diseases such as diabetes mellitus, hypertension and malignancies are new challenges among COVID-19 patients [10, 11]. Several studies have shown that coinfection with *Mtb* or HIV is associated with disease severity among COVID-19 patients [12, 13].

On July 30, 2021, the first local patient was confirmed to be infected with SARS-CoV-2 in Zhengzhou, which led to a new wave of infection. After population screening, active contact tracing and centralized quarantine, 166 patients diagnosed with Delta variant infection were sent to a specific hospital for intensive therapy. Epidemiological evidence suggests that most local patients visit the Sixth People Hospital of Zhengzhou, which is an infectious disease hospital. Hence, these cases provided a unique opportunity to understand more about patients infected with the Delta variant and coinfected with *Mtb* or HIV. We also collected clinical data for patients infected with the Omicron variant in January 2022 to explore differences in clinical characteristics between patients with Delta and Omicron infections.

## Materials and methods

### Data sources

**Fig. 1:**
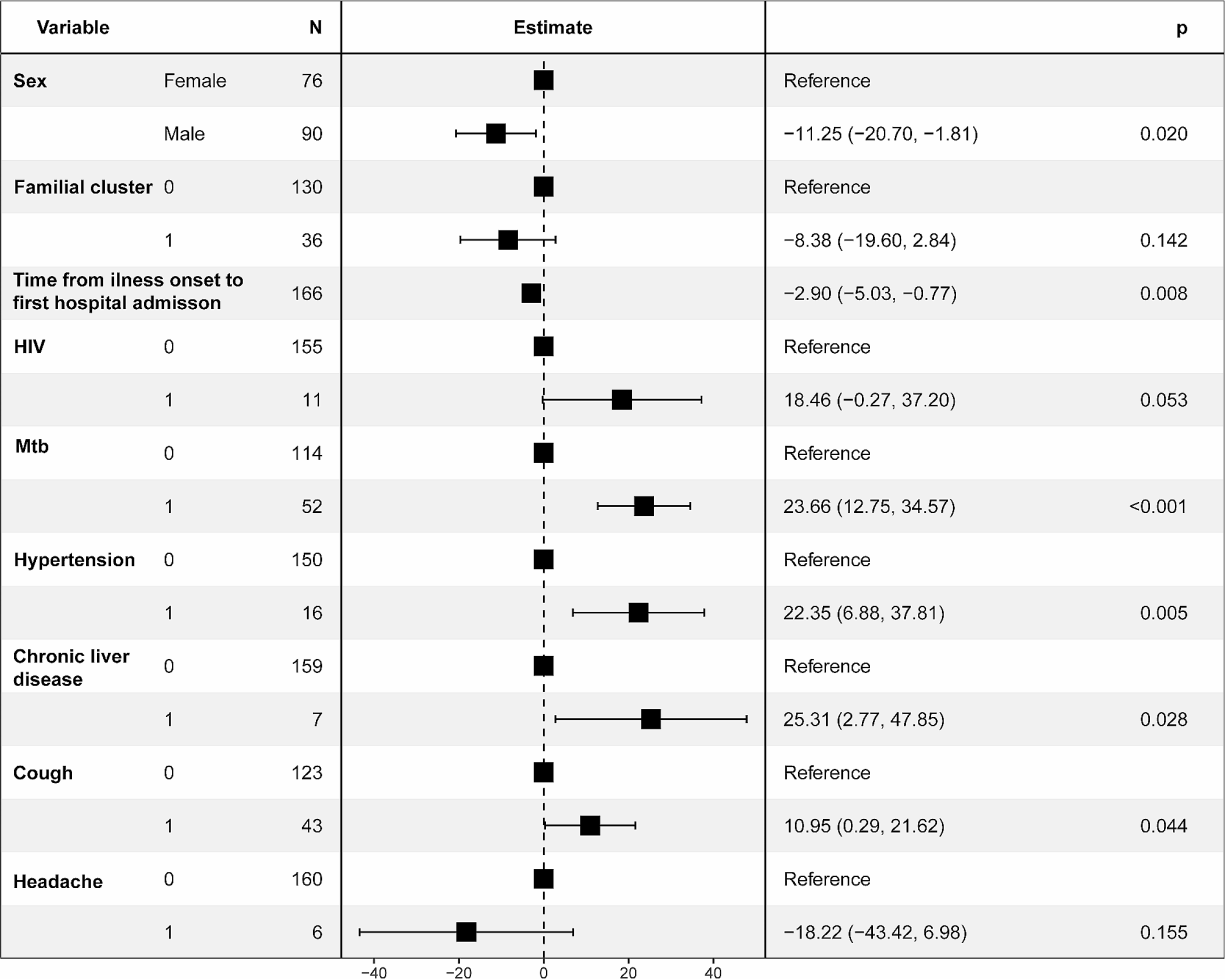
Forest plot of 166 Delta-infected patients. The seven variables were selected by a linear regression model according to the variables in Table 1.

We performed a retrospective study concentrating on the clinical and immunological characteristics of 166 patients diagnosed with the Delta variant (B.1.617) from 30 July 2021 to 30 November 2021 in Zhengzhou and 384 patients diagnosed with the Omicron variant (BA.1) from January 8, 2022 to February 13, 2022 in Anyang. According to the variables in Table 1, seven variables affecting the LOS were screened by a linear regression model; these included sex, familial cluster, time from illness onset to first hospital admission, HIV, *Mtb*, hypertension, chronic liver disease, cough and headache (Fig. 1). Thus, we separated 166 Delta-infected patients into three groups according to coinfections, namely, 103 Delta-infected patients (Delta group), 52 Delta-infected patients coinfected with *Mtb* (*Mtb* group), and 11 Delta-infected patients coinfected with HIV (HIV group). A total of 384 patients infected with Omicron were included in the Omicron group.

**Table 1:** Demographic and baseline clinical characteristics of patients in the four groups

	Delta group (*n* = 103)	Mtb group (*n* = 52)	HIV group (*n* = 11)	Omicron group (*n* = 384)	P1-value	P2-value	P3-value	P4-value
Characteristics								
Median (interquartile) age (years)	41 (28–57)	49 (27–62)	47 (32–51)	18 (17–34)	0.880	0.331	0.715	0
Age groups (years)					0.25	1.0	0.443	0
≤ 18	11 (11%)	2 (4%)	1 (9%)	235 (61%)	··	··	··	··
19–40	40 (39%)	22 (42%)	2 (18%)	72 (19%)	··	··	··	··
41–65	39 (38%)	18 (35%)	8 (73%)	58 (15%)	··	··	··	··
≥ 66	13 (13%)	10 (19%)	0	19 (5%)	··	··	··	··
Male sex	45 (44%)	37 (71%)	8 (73%)	162 (42%)	0.0012	0.066	0.0495	0.784
Familial cluster	28 (27%)	8 (15%)	0	166 (46%)	0.10	0.10	0.371	0.001
Coexisting conditions	34 (33%)	17 (33%)	6 (55%)	39 (10.2%)	0.935	0.246	0.306	< 0.0001
Diabetes	14 (14%)	10 (19%)	5 (45%)	10 (3%)	0.36	0.0029	0.039	0
Hypertension	12 (12%)	3 (6%)	1 (9%)	23 (6%)	0.38	1.0	0.546	0.048
Cardiovascular disease	8 (8%)	3 (6%)	0	6 (1.6%)	0.90	1.0	1.0	0.003
Chronic obstructive pulmonary disease	0	1 (2%)	0	0	0.34	··	1.0	··
Number of vaccination doses					< 0.0001	0.001	0.078	< 0.0001
0	40 (39%)	34 (65%)	11 (100%)	2 (1%)	··	··	··	··
1	7 (7%)	16 (31%)	0	11 (3%)	··	··	··	··
2	53 (51%)	2 (4%)	0	346 (90%)	··	··	··	··
3	1 (1%)	0	0	21 (5%)	··	··	··	··
Fever	36 (35%)	11 (21%)	3 (27%)	96 (25%)	0.21	0.86	1.0	0.038
Highest temperature, °C					0.13	0.86	1.0	0.083
< 37.3	67 (65%)	41 (79%)	8 (73%)	283 (74%)	··	··	··	··
37.3–38	17 (17%)	5 (10%)	1 (9%)	48 (12%)	··	··	··	··
> 38	19 (18%)	6 (11%)	2 (18%)	53 (14%)	··	··	··	··
Cough	26 (25%)	14 (27%)	3 (27%)	240 (63%)	0.82	1.0	1.0	0
Expectoration	16 (16%)	5 (10%)	1 (9%)	112 (29%)	0.31	0.90	1.0	0.005
Myalgia or fatigue	9 (9%)	19 (36%)	4 (36%)	30 (6%)	0.0005	0.098	1.0	0.002
Nasal congestion	7 (7%)	9 (17%)	0	96 (25%)	0.042	1.0	0.31	0
Loss of smell and taste	2 (2%)	5 (10%)	2 (18%)	4 (1%)	0.078	0.046	0.769	0.816
Headache	3 (3%)	3 (6%)	0	7 (2%)	0.67	1.0	1.0	0.763
Dyspnea	0	0	0	3 (0.8%)	··	··	··	1
Severity score					0.706	0.519	0.788	< 0.0001
4	84 (81%)	40 (77%)	8 (73%)	380 (99%)	··	··	··	··
5	17 (17%)	10 (19%)	3 (27%)	4 (1%)	··	··	··	··
9	2 (2%)	2 (4%)	0		··	··	··	··
The incubation period (days)	3 (2–7) (*n* = 81)	2 (1–3) (*n* = 48)	4 (2–6) (*n* = 8)	2 (1–2) (*n* = 97)	0.31	0.0004	0.815	0
Time from illness onset to first hospital admission (days)	2.5 (0–5.0)	5 (4–5)	3 (2–4)	2 (1–3) (*n* = 362)	0.003	< 0.0001	0.999	0.840

*P1*-value comparing the Delta group and *Mtb* group, *P2*-value comparing the Delta group and HIV group and *P3*-value comparing *Mtb* group and HIV group are from χ², or ANOVA. *P4*-value comparing the Delta group and Omicron group is from χ², Fisher’s exact test, or Mann-Whitney U test. LOS, length of stay

Since COVID-19 broke out, the government has implemented stringent screening measures, including multiple nucleic acid tests for residents in the city, especially those who visited the sixth people’s hospital in Zhengzhou or had contact with infected persons after 5 July 2021. Epidemiological, clinical, laboratory, and immunological characteristics and treatment and outcome data were obtained with standard data collection forms from electronic medical records.

This study was approved by the Institutional Review Board of the First Affiliated Hospital of Zhengzhou University (L2021-Y266-002) and was conducted according to the Declaration of Helsinki and Good Clinical Practice. The requirement for informed consent was waived by the Institutional Review Board of the First Affiliated Hospital of Zhengzhou University because of the urgent need to collect data on this new pathogen.

### Laboratory confirmation

Positive high-throughput sequencing or real-time reverse transcription polymerase chain reaction (RT–PCR) of nasal and pharyngeal swab specimens confirmed the diagnosis of SARS-CoV-2. By sequencing the viral RNA gene and comparing the sequence with the known reference sequence of the virus, all patients were found to have Delta variant (B.1.617) infection. Nasopharyngeal swabs were collected by skilled medical staff at the same hospital following strictly standardized procedures. Laboratory investigations, including complete blood analysis and serum biochemistry, were performed on admission.

### Lymphocyte subpopulation measurement

Samples of EDTA anticoagulated peripheral blood (2 mL) were collected from patients with the Delta variant on admission. All the samples were tested within 6 h. Briefly, CD3+/CD4+/CD8 + T-cell, CD19 + B-cell, and CD16 + CD56 + NK-cell counts (cells/µL) were measured by multiple-color flow cytometry with human monoclonal anti-CD3-fluorescein isothiocyanate (FITC), anti-CD8-phycoerythrin (PE), anti-CD45-PerCP, anti-CD4-allophycocyanin (APC), anti-CD16-PE, anti-CD56-PE, and anti-CD19-APC antibodies (BD Multitest) according to the manufacturer’s instructions. The cells were analyzed on a BriCyte E6 flow cytometry system (Mindray).

### Cytokine measurement

To characterize the effect of the Delta variant on the production of cytokines at admission, plasma cytokines (IL2, IL4, IL6, IL8, IL10, IL12p70, IL17A, IFNr and TNFα) were measured using a Human High Sensitivity T-Cell Magnetic Bead Panel and the BriCyte E6 system (Mindray, Shenzhen, China) in Delta-infected patients according to the manufacturer’s instructions.

### Definitions

We defined the incubation period as the date from contact to presentation. The incubation period was estimated for patients who were able to provide the specific date of close contact with a person who was confirmed or suspected of having SARS-CoV-2 infection. Disease duration was defined as the first exposure to the sources of infection for two consecutive negative nucleic acid tests. The length of stay (LOS) was defined as the time from admission to discharge. The NLR represents the neutrophil-to-lymphocyte ratio, while the PLR represents the platelet-to-lymphocyte ratio.

### Statistical analysis

Given that the original source of the Delta variant outbreak was an infectious disease hospital, we divided the Delta infection cohort into only Delta-infected patients, patients coinfected with *Mtb*, and patients coinfected with HIV. Patients coinfected with *Mtb* were divided into 49 nonchronic patients and three chronic patients according to disease duration. Quantitative data with a skewed distribution are summarized as medians and interquartile ranges, and quantitative data with a normal distribution are summarized as the means ± standard deviations. For classified variables, the percentages of patients in each category were calculated. We used ANOVA and the chi-square test to compare the clinical characteristics among only Delta-infected patients, patients coinfected with *Mtb*, and patients coinfected with HIV. For the Delta and Omicron groups, the Mann‒Whitney U test, the chi-square test or Fisher’s exact test (as appropriate) was used. A *P* value less than 0.05 indicated statistical significance. All analyses were performed with SPSS software for Windows, version 26.

## Results

### Epidemiological characteristics

There were more males among those with Delta coinfected with *Mtb* (71%) or HIV (73%; Table 1). In the Delta group, the median age was 41 years (interquartile range 28–57 years; Table 1). Twenty-eight (27%) patients were associated with family clusters, which differed significantly from those of the Omicron group. In the Omicron group, the proportions of patients aged less than 18 years (235, 61%) and who completed two doses of the COVID-19 vaccine (346, 90%) were much greater than those in the other three groups. In the *Mtb* and HIV groups, the median ages were 49 years (IQR 27–62 years) and 47 years (IQR 32–51), respectively. Eight out of 52 cases (15%) in the *Mtb* group were related to family clusters, but none were related to the HIV group. Only four patients in the two groups completed two doses of the vaccine. In the four groups, more than half of the patients had a severity score of 4 according to the WHO Clinical Progression Scale.

### Clinical features

In the Delta group, twenty-four (23%) patients had one or more underlying diseases—14 (14%) had diabetes, twelve (12%) had hypertension, eight (8%) had cardiovascular disease, and none had chronic obstructive pulmonary disease (Table 1). However, in the *Mtb* and HIV groups, seventeen (33%) and six (55%) individuals, respectively, had underlying diseases—ten (19%) and five (45%) had diabetes, three (6%) and one (9%) had hypertension, three (6%) and zero had cardiovascular disease, and one (2%) and none had chronic obstructive pulmonary disease, respectively. In the Omicron group, the proportion of patients with underlying disease was much lower than that in the other three groups. In the Delta group, the most common symptoms at admission were fever (36 [35%]), cough (26 [25%], and expectoration (16 [16%]), which was consistent with the symptoms of the other three groups. In contrast, high rates of myalgia or fatigue were observed in the *Mtb* and HIV groups. Among the four groups, the Omicron group presented the shortest incubation period (2.0 days [IQR 1.0–2.0 days]) and the shortest time from symptom onset to first hospital admission (2.0 days [IQR 1.0–3.0 days]).

### Laboratory abnormalities

**Fig. 2:**
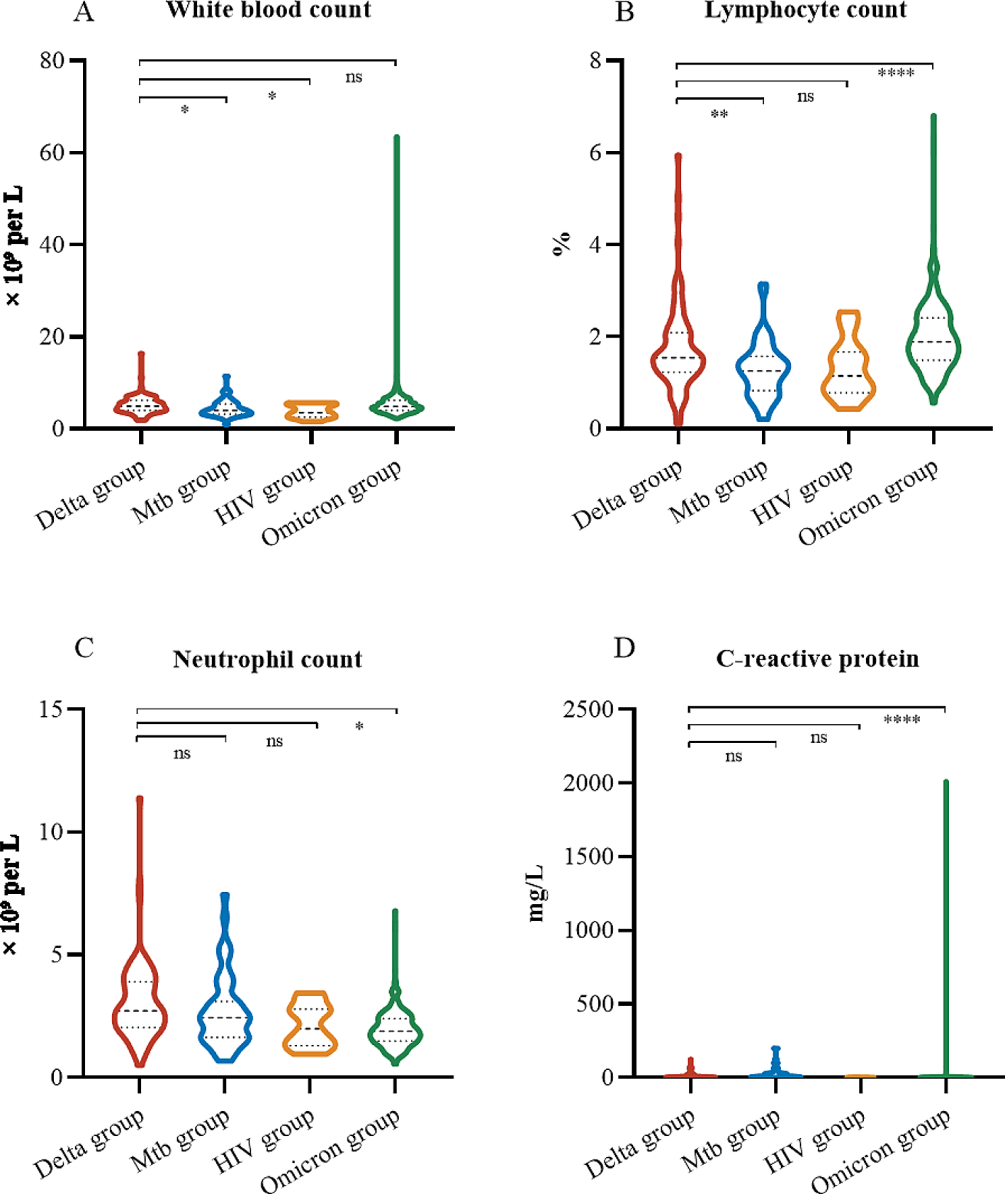
Main laboratory findings in the four groups of patients upon admission to the hospital. (**A**) Comparison of white blood cell counts among the four groups. (**B**) Comparison of lymphocyte counts among the four groups. (**C**) Comparison of C-reactive protein levels among the four groups. (**D**) Comparison of neutrophil counts among the four groups. *, *P* <.05; **, *P* <.01; ***, *P* <.001; ****, *P* <.0001; ns, not significant

Compared with those in the Delta group, both in the *Mtb* and HIV group, the white blood cell was lower (4.10 [3.20–5.40] and 3.57 [2.58–5.46]), and blood analysis revealed lymphopenia (25 [48%] and 5 [45%]; Table 2; Fig. 2A). In the *Mtb* group, compared with those in the Delta group, lymphocyte counts were lower, but the lactate dehydrogenase level was increased (Fig. 2B). Significantly, 25 of the 52 (48%) patients presented leukopenia (white blood cell count < 4 × 10^9^/L). Among the three groups, there were no significant differences in the neutrophil or C-reactive protein levels (Fig. 2C and D). In the Omicron group, the neutrophil count, lactate dehydrogenase level, C-reactive protein level and NLR decreased, while the lymphocyte count, platelet count and PLR increased.

**Table 2:** Laboratory findings for the 4 groups of patients upon admission to the hospital

	Delta group (*n* = 103)	Mtb group (*n* = 52)	HIV group (*n* = 11)	Omicron variant (*n* = 384)	P1-value	P2-value	P3-value	P4-value
White blood cell, × 10^9^/L	4.98 (4.00-6.21)	4.10 (3.20–5.40)	3.57 (2.58–5.46)	4.91 (4.06–6.22)	0.017	0.023	0.336	0.935
< 4	25 (24%)	25 (48%)	6 (55%)	87 (23%)	0.0028	0.074	0.750	0.729
4–10	76 (74%)	26 (50%)	5 (45%)	293 (76%)	··	··	··	··
> 10	2 (2%)	1 (2%)	0	4 (1%)	··	··	··	··
Neutrophil, × 10^9^/L	2.72 (2.05–3.91)	2.5 (1.6–3.1)	1.99 (1.31–2.79)	2.43 (1.82–4.33)	0.181	0.055	0.25	0.011
Lymphocyte, × 10^9^/L	1.54 (1.23–2.10)	1.26 (0.83–1.58)	1.15 (0.78–1.67)	1.89 (1.49–2.41)	0.001	0.113	0.89	< 0.0001
<1.0	15 (15%)	17 (33%)	5 (45%)	15 (15%)	0.0085	0.032	0.647	0.001
Eosinophil, × 10^9^/L	0.08 (0.03–0.14)	0.065 (0.02–0.15)	0.07 (0.03–0.17)	205 (173-241.5)	0.658	0.007	0.018	< 0.0001
Platelet, × 10^9^/L	164 (119–204)	144.5 (107.5-197.8)	155 (91–165)	175 (144–193)	0.549	0.484	0.718	< 0.0001
< 100	13 (13%)	9 (17%)	3 (27%)	19 (6%)	0.89	0.94	0.732	0
≥ 100	91 (87%)	43 (83%)	7 (64%)	315 (94%)	··	··	··	··
Haemoglobin, g/L	128 (119–146)	121 (94–143)	128 (107–140)	143 (131–159)	0.315	0.736	0.846	0.018
Prothrombin time, s	11.8 (11.4–12.4)	11.8 (11.4–12.8)	11.8 (11.3–11.8)	11.7 (10.9–12.5)	0.528	0.488	0.32	0.0003
D-dimer, mg/L	0.33 (0.19–0.60)	0.3 (0.2–0.8)	0.60 (0.23–1.15)	0.04 (0.02–0.09)	0.538	0.695	0.945	0
≥ 1.0	10 (10%)	9 (17%)	3 (27%)	2 (0.5%)	0.76	0.72	0.732	0.91
Alanine aminotransferase, U/L	17 (12–29)	17 (11-24.5)	17 (12–54)	17 (12–26)	0.513	0.436	0.683	0
Aspartate aminotransferase, U/L	23 (17–30)	26.5 (20.0-45.5)	20 (15–36)	24 (21–30)	0.013	0.998	0.066	0
Albumin, g/L	42.68 (40.16–45.60)	40.5 (35.3–43.9)	42.4 (38.6–45.3)	43 (41.1–45.1)	0.001	0.932	0.114	0.699
Total Bilirubin, µmol/L	11.2 (8.48–15.03)	9.2 (6.35–11.28)	8.6 (5.2–11.8)	8.5 (6.0-12.4)	0.594	0.580	0.798	0.018
Blood urea nitrogen, µmol/L	4.44 (3.82–5.21)	4.9 (4.2–6.4)	5.30 (4.88–6.14)	3.9 (3.5–4.3)	0.252	0.516	0.970	0.203
Creatinine, µmol/L	59.5 (49-71.5)	61.0 (52.0-72.3)	71 (49–80)	69 (62–78)	0.138	0.126	0.48	0
≥ 133	2 (2%)	3 (6%)	2 (18%)	58 (15%)	0.59	0.13	0.207	0
Creatinine kinase, U/L	61.5 (38.7–91.7)	37.5 (24.2–76.5)	47.5 (25.6-120.6)	86 (63–116)	0.277	0.948	0.488	0.044
Lactate dehydrogenase, U/L	200 (173–243)	224 (175–269)	213 (165–271)	158 (140–180)	0.026	0.872	0.317	< 0.0001
≥ 250	25 (24%)	17 (33%)	3 (27%)	8 (2%)	0.031	0.90	1.0	0
C-reactive protein, mg/L	3.9 (1.5–18.5)	10.9 (3.3–29.8)	1.55 (0.85–2.53)	1.57 (0.14–4.20)	0.204	0.624	0.258	< 0.0001
> 10	36 (35%)	24 (46%)	0	28 (7%)	0.27	0.83	0.11	0
NLR	1.73 (1.26–2.63)	2.04 (1.38–3.40)	1.49 (1.08–2.55)	1.31 (0.91–1.81)	0.842	0.457	0.417	< 0.0001
PLR	99.4 (72.66-135.21)	118.89 (80.2-192.96)	139.53 (62.60-178.41)	106.53 (83.22-143.87)	0.690	0.974	0.814	0
Pneumonia	63 (61%)	38 (73%)	11 (100%)	116 (30%)	0.14	0.033	0.17	0
Bilateral involvement of chest radiographs	66 (50%)	32 (62%)	7 (64%)	51 (13%)	0.22	0.43	1.0	0

*P1*-value comparing the delta group and TB group, *P2*-value comparing the delta group and HIV group and *P3*-value comparing *Mtb* group and HIV group are from χ², or ANOVA. *P4*-value comparing the delta group and omicron group is from χ², Fisher’s exact test, or Mann-Whitney U test. NLR, neutrophil-lymphocyte ratio; PLR, planet-lymphocyte ratio

**Fig. 3:**
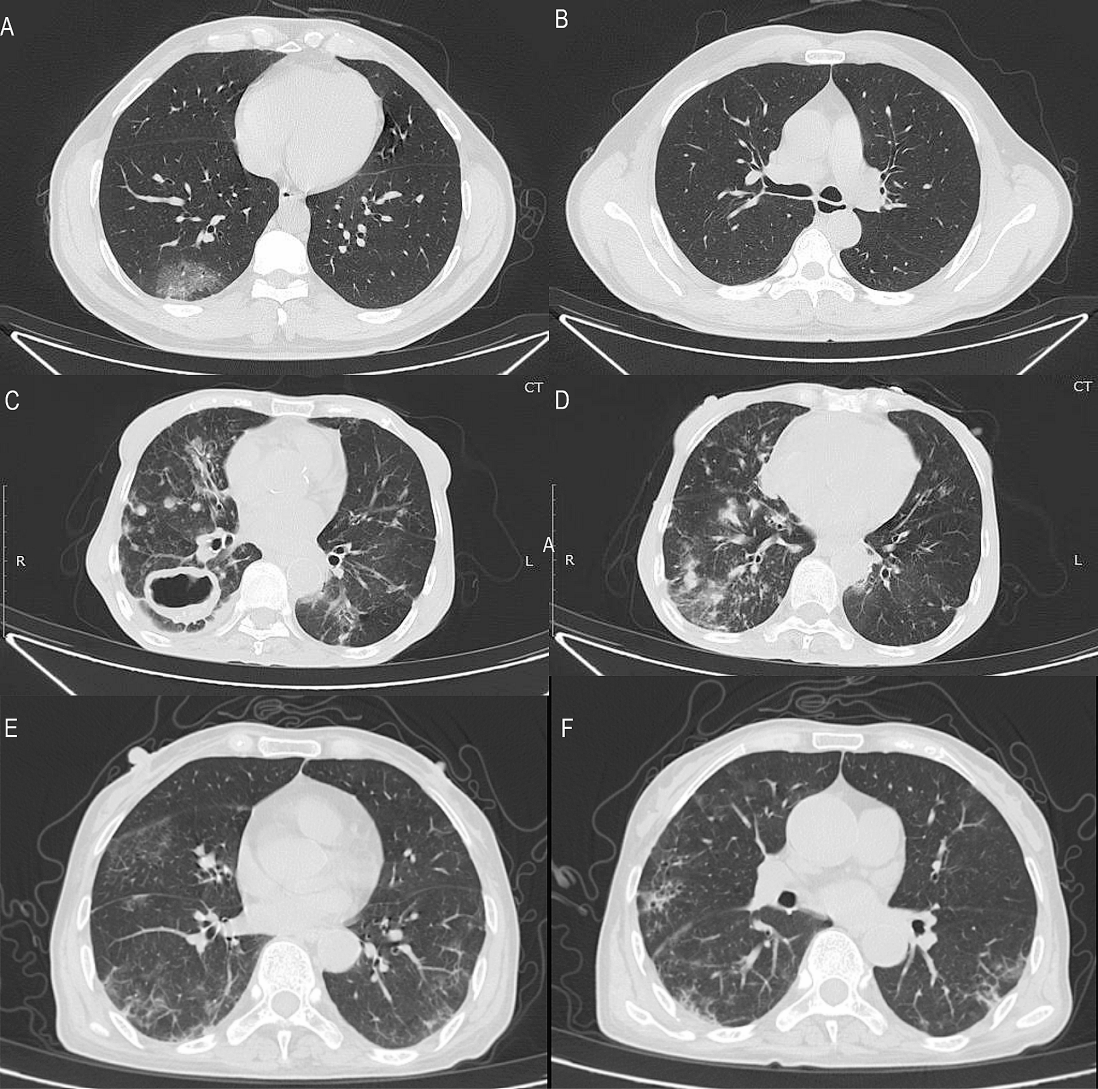
Representative chest CT images. (**A** and **B**) Transverse chest CT image of a 48-year-old man infected with Delta only showing patchy ground-glass opacities in the inferior lobe of the right lung five days after symptom onset. (**C** and **D**) Transverse chest CT images from a 66-year-old woman coinfected with *Mtb* showing tuberculous cavities and bilateral multiple patchy shadows on day six after symptom onset. (E and F) Transverse chest CT images from a 58-year-old woman coinfected with HIV showed multiple patchy, streaky ground-glass opacities in both lungs on day two after symptom onset.

On admission, except for those in the Omicron group, more than half of the patients in the three groups, particularly those in the HIV group (11, 100%), had abnormal chest CT findings. The typical findings on chest CT images of Delta-infected patients at admission were signs of pneumonia (Fig. 3A and B). Delta-infected patients with *Mtb* typically exhibited tuberculous cavities and bilateral patchy shadows on chest CT (Fig. 3C and D). Representative chest CT scans of Delta-infected patients with HIV showed multiple lobular areas of consolidation and bilateral ground-glass opacities (Fig. 3E and F).

### Lymphocyte subpopulations and cytokines

**Fig. 4:**
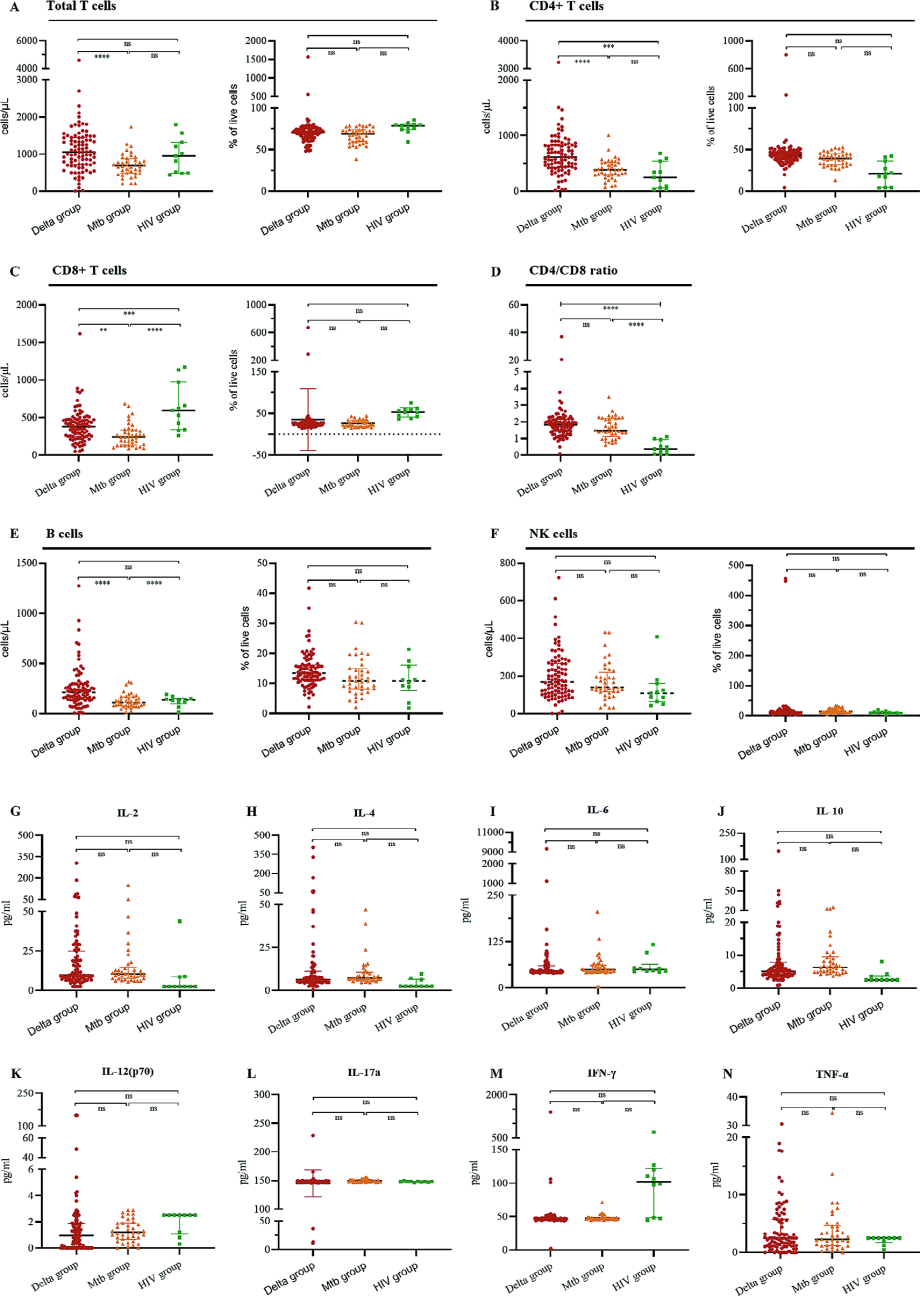
Lymphocyte subpopulations and plasma cytokine levels in three groups of Delta-infected patients upon admission to the hospital. (**A**-**F**) Comparison of lymphocyte subpopulations between Delta-infected patients and Delta-infected patients and between *Mtb*- or HIV-infected patients. (**G**-**N**) Comparison of cytokine levels between Delta-infected patients and Delta-infected patients with *Mtb* or HIV. The vertical axis of each medium differs to accommodate extreme variations in the normally distributed range of lymphocyte subpopulations and cytokine concentrations. The data are presented as medians and interquartile ranges. IL = interleukin; IFN-γ = interferon γ; TNF-α = tumor necrosis factor α; *, *P* <.05; **, *P* <.01; ***, *P* <.001; ****, *P* <.0001; ns, not significant

We found that the total T-cell (686.0 [485.3-875.5]), CD4 + T-cell (382.5 [290.5-511.8], CD8 + T-cell (243.0 [134.0-335.5]) and B-cell (108.0 [74.0-165.75]) counts were significantly lower in the *Mtb* group than in the Delta group (Fig. 4A-F; Table 3). In the HIV group, compared with those in the Delta group, the counts of CD4 + T cells (246 [56–540]) and B cells (136 [98–153]) were lower, while the number of CD8 + T cells (595 [340–973]) was greater. In addition, the percentage of CD8 + T cells was markedly greater in the HIV group than in the Delta group. For cytokines, there were no significant differences among the three groups (Fig. 4G-N; Table 3).

**Table 3:** Lymphocyte subpopulation and cytokine levels of Delta-infected patients at admission to the hospital

	Delta group (*n* = 103)	Mtb group (*n* = 52)	HIV group (*n* = 11)	P1-value	P2-value	P3-value
Lymphocyte subpopulations						
Total T cells, per µl	1051.0 (712.0-1441.5)	686.0 (485.3-875.5)	947 (484–1320)	< 0.0001	0.715	0.299
Total T cells, %	70.76 (65.53–74.99)	68.94 (59.66–74.05)	78.61 (74.10-80.38)	0.335	0.727	0.832
CD4^+^ T cells, per µl	610.0 (428.5–830.0)	382.5 (290.5-511.8)	246 (56–540)	< 0.0001	0.0008	0.375
CD4^+^ T cells, %	42.96 (37.61–47.61)	39.19 (31.64–44.99)	21.04 (4.28–36.26)	0.266	0.145	0.453
CD8^+^ T cells, per µl	381.0 (250.5-474.5)	243.0 (134.0-335.5)	595 (340–973)	0.0027	0.0004	< 0.0001
CD8^+^ T cells, %	24.26 (21.13–29.54)	23.73 (19.10-31.35)	52.68 (42.66–60.48)	0.407	0.367	0.191
CD4/CD8 ratio	1.82 (1.41–2.15)	1.48 (1.12–2.20)	0.37 (0.07–0.94)	0.243	0.084	0.330
B cells, per µl	211.0 (138.5-295.5)	108.0 (74.0-165.75)	136 (98–153)	< 0.0001	< 0.0001	1.0
B cells, %	13.42 (11.04–16.17)	10.76 (8.05–14.87)	10.78 (7.57–16.16)	0.062	0.114	0.627
NK cells, per µl	168 (107-267.5)	140.0 (115.8-219.3)	108 (64–180)	0.231	0.074	0.311
NK cells, %	11.76 (8.04–16.96)	13.22 (11.11–23.27)	9.22 (7.08–11.34)	0.497	0.443	0.734
Cytokines						
IL-2	9.75 (7.13–24.95)	10.45 (7.65–14.63)	2.5 (2.5–8.65)	0.424	0.193	0.430
IL-4	6.25 (5.00-11.18)	7.1 (6.3–10.6)	2.5 (2.5–6.43)	0.270	0.297	0.706
IL-6	44.75 (42.48–59.78)	49.40 (43.85–62.25)	49.3 (45.38-64)	0.482	0.683	1.0
IL-10	5.1 (3.9–7.8)	6.35 (4.90–9.45)	2.5 (2.5–3.78)	0.625	0.179	0.318
IL-12p70	0.95 (0.08–1.90)	1.2 (0.65–1.93)	2.5 (1.1–2.5)	0.302	0.624	0.918
IL-17 A	147.7 (147-148.5)	148.6 (147.5-151.6)	148.1 (147.3-148.5)	0.221	0.640	0.818
IFN-γ	46.4 (45.6–48.0)	47.20 (46.18–48.93)	102.4 (48-121.9)	0.745	0.485	0.354
TNF-a	2.45 (1.10–5.78)	2.25 (1.18–4.65)	2.5 (1.7–2.5)	0.998	0.252	0.281

*P1*-value comparing the delta group and *Mtb* group, *P2*-value comparing the delta group and HIV group and *P3*-value comparing *Mtb* group and HIV group are from χ², or ANOVA

### Treatment and clinical outcomes

**Fig. 5:**
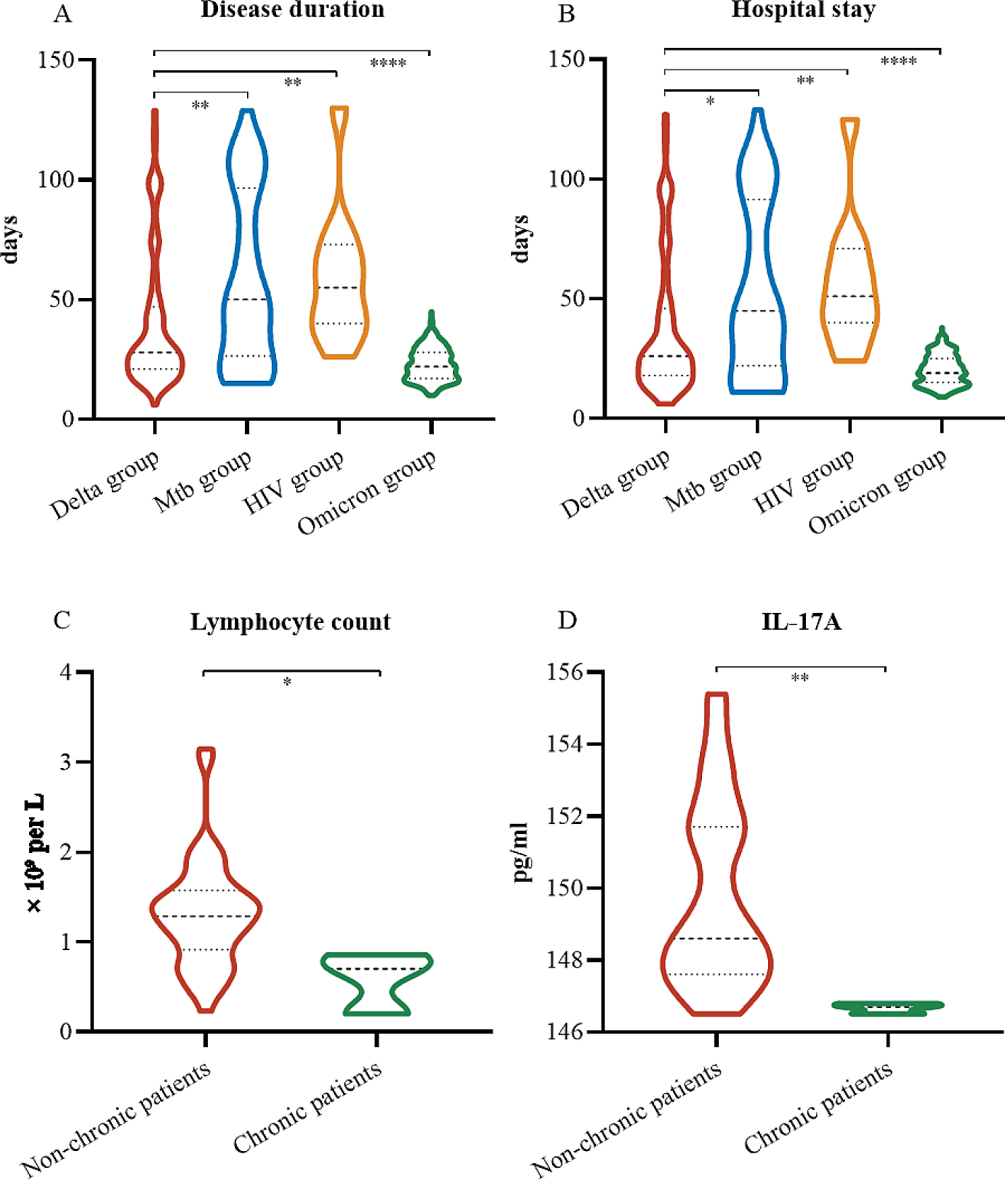
Disease duration and hospital stay for three groups of Delta-infected patients. (**A**) Disease duration in the four groups. (**B**) LOS in the four groups. (**C**) Comparison of the serum levels of IL-17 A between the nonchronic and chronic groups in the *Mtb* group. (**D**) Comparison of lymphocyte counts between the nonchronic and chronic groups in the *Mtb* group. *, *P* <.05; **, *P* <.01; ***, *P* <.001; ****, *P* <.0001; ns, not significant

Among all the patients, just twenty were admitted to an intensive care unit. Few patients developed complications, including secondary infection and shock (Table 4). In the *Mtb* group, just one (2%) patient developed secondary infection, and two (4%) went into shock (Table 4). Isoniazid, rifampicin, ethambutol and pyrazinamide are used in anti-tuberculosis treatment. All HIV patients received antiretroviral treatment. Among the three groups of patients, almost all patients received traditional Chinese medicine (TCM) treatment, which is based on pattern differentiation and the principle of personalized diagnosis and treatment. Overall, 35 (34%), 37 (71%), nine (82%) and 143 (37%) patients received immunotherapy, including thymalfasin and vitamin C, while 16 (16%), 35 (67%) and five (45%) patients, respectively, received antiviral therapy, including arbidol, lopinavir and ritonavir. Only five (3%) patients received plasma treatment from recovered patients. All patients were discharged, and no patients died. Compared with that in the Delta group (28 days [IQR 21–47 days]), the disease duration in the *Mtb* group or the HIV group was longer (50 [IQR 26.5–96.5] or 55 [IQR 40–73]; Fig. 5A). Similarly, compared with that in the Delta group (26 [interquartile range (IQR) 18–46]), the LOS in patients coinfected with *Mtb* or HIV was longer (45 [IQR 22-91.5] or 51 [IQR 40–71]; Fig. 5B). The lack of suitability for discharge was in accordance with the absence of clinical features, the improved evidence from chest CT and the use of two negative nucleic acid detection methods (24 h apart).

**Table 4:** Treatments and outcomes of patients in the four groups

	Delta group (*n* = 103)	Mtb group (*n* = 52)	HIV group (*n* = 11)	Omicron group (*n* = 384)	P1-value	P2-value	P3-value	P4-value
Admission to intensive care unit	10 (10%)	8 (15%)	2 (18%)	0	0.30	0.72	1.0	< 0.0001
Secondary infection	0	1 (2%)	0	1 (0.3%)	0.34	**··**	1.0	1
Shock	1 (1%)	2 (4%)	0	0	0.54	1.0	1.0	0.211
Treatment								
TCM treatment	93 (90%)	47 (90%)	11 (100%)	381 (99%)	0.99	0.60	0.58	< 0.0001
Immunotherapy	35 (34%)	37 (71%)	9 (82%)	143 (37%)	< 0.0001	0.0056	0.73	0.542
Antiviral treatment	16 (16%)	35 (67%)	5 (45%)	8 (2%)	< 0.0001	0.043	0.31	< 0.0001
Antibiotics	16 (16%)	40 (77%)	2 (18%)	11 (3%)	< 0.0001	1.0	0.001	< 0.0001
Heparin	26 (25%)	6 (12%)	6 (55%)	33 (9%)	0.16	1.0	0.58	0
Plasma of recovered patients	2 (2%)	2 (4%)	1 (9%)	NA	0.87	0.16	0.44	··
Corticosteroid and gamma globulin	0	2 (4%)	0	NA	0.11	0.0021	0.44	··
Glucocorticoids alone	0	8 (15%)	0	0	< 0.0001	··	0.37	··
Continuous renal-replacement therapy	0	3 (6%)	0	0	0.014	··	1.0	··
Oxygen support								
Nasal cannula	17 (17%)	7 (13%)	2 (18%)	4 (1%)	0.62	1.0	1.0	< 0.0001
Non-invasive ventilation or high-flow nasal cannula	2 (2%)	5 (9%)	1 (9%)	0	0.078	0.27	1.0	< 0.0001
Invasive mechanical ventilation	2 (2%)	2 (4%)	0	0	0.87	1.0	1.0	0.014
Invasive mechanical ventilation and ECMO	2 (2%)	2 (4%)	0	0	0.87	1.0	1.0	0.014
Prognosis								
Hospitalisation	0	0	0	0	··	··	··	··
Discharge	103 (100%)	52 (100%)	11 (100%)	384 (100%)	··	··	··	··
Death	0	0	0	0	··	··	··	··
LOS, days	26 (18–46)	45.0 (22.0-91.5)	51 (40–71)	19 (15–25)	0.018	0.008	0.993	< 0.0001
Disease duration	28 (21–47)	50 (26.5–96.5)	55 (40–73)	22 (17–28)	0.007	0.006	0.990	< 0.0001

TCM treatment, traditional Chinese medicine treatment; LOS, length of stay

### Delta-infected patients without vaccinations

In the Delta group, more than half of the patients had been vaccinated, which may have affected disease duration and severity. Therefore, we analyzed the epidemiology, laboratory test results, lymphocyte subpopulations, cytokine levels, prognoses and outcomes of unvaccinated patients in the Delta, *Mtb* and HIV groups separately. In the *Mtb* group, myalgia or fatigue, nasal congestion and loss of smell and taste occurred at a greater rate than in the Delta group (Table S1). In the HIV group, only loss of smell and taste frequently occurred. According to our laboratory test results, only the white blood cell count was lower in the HIV group than in the Delta group (Table S2). The differences in the remaining parameters were not statistically significant among the three groups. Compared with those in the Delta group, the total T-cell (719.0 [481.5-911.5], CD4 + T-cell (411 [239.5-522.3], and B-cell (104 [78.0-174.8]) counts were significantly lower in the *Mtb* group (Table S3). In the HIV group, compared with those in the Delta group, the counts of CD4 + T cells (246 [56–540]) and B cells (136 [98–153]) were lower, while the number of CD8 + T cells (595 [340–973]) was greater. For cytokines, only IL-2 and IL-10 were lower in the HIV group than in the *Mtb* group (Table S3). There was no significant difference in the LOS or disease duration among the three groups (Table S4).

### Pediatric patients (under 18 years old) in the Delta and Omicron groups

In total, 61% of the pediatric patients in the Omicron group (under 18 years old) were diagnosed with more mild symptoms. Thus, it was necessary to analyze individuals under 18 years old from the Delta and Omicron groups separately. In the Omicron group, cough was more common than in the Delta group (Table S5). D-dimer levels were significantly greater in the Delta group (4.02 ± 10.87) than in the Omicron group (0.04 [0.01–0.09]; Table S6). The LOS and disease duration were longer in the Delta group than in the Omicron group (Table S7).

### Chronic disease course under double infection by SARS-CoV-2 and *M. tuberculosis*

In the *Mtb* group, three (6%) patients had a chronic disease course of more than four months. We classified these 52 patients into two groups: 49 patients in the chronic group and 3 patients in the nonchronic group. We compared clinical characteristics, and the results indicated that the plasma concentration of IL-17 A and lymphocyte count were lower in the chronic group than in the nonchronic group (Fig. 5C and D; Tables S8-S10). Reduced expression of IL-17 A suggested potential dysfunction of the Th17 cell response during the chronic course of the disease.

## Discussion

According to two different studies from Canada and Scotland, Delta-infected patients were more likely to be hospitalized than were those diagnosed with Alpha- or the original virus that caused COVID-19 [14], which suggested that the Delta variant might cause more severe disease in unvaccinated individuals than was the previous SARS-CoV-2 variant. Nevertheless, in previous studies, the clinical features of patients with the Delta variant differed from those of patients with the Wuhan variant [15, 16], with mild clinical symptoms and no mortality, which may be attributed to the popularization of COVID-19 vaccination and the improvement of treatment in China.

Here, we found that SARS-CoV-2 patients coinfected with *Mtb* or HIV exhibited a longer disease duration and LOS with decreased B-cell and CD4 + T-cell counts, suggesting that prolonged viral shedding related to immune function was involved. The immune response is the body’s main protection against viral infections. The number of lymphocytes affects the strength of the immune response and thereby the severity of the disease [17]. Both *M. tuberculosis* and HIV can interfere with the immune system. Studies have shown that T cells are reduced during infection, which might contribute to the progression of the disease [18]. Viral replication under multiple conditions (i.e., coinfections, state of immunosuppression) may lead to intrahost evolution and the emergence of new variants, impairing antibody neutralization and leading to prolonged viral shedding, as demonstrated in patients with HIV viraemia [19, 20]. Notably, in our study, the lymphocyte, B-cell and CD4 T-cell counts were significantly lower in the *Mtb* and HIV groups than in the control group. These findings indicate increased disease severity and decreased immune function, which are detrimental to infection control. In addition, 51% of individuals in the Delta group received 2 doses of the COVID-19 vaccine, which affected disease duration and severity. Thus, unvaccinated Delta-infected patients were compared separately. In the *Mtb* and HIV groups, the number of B cells and CD4 + T cells was lower than that in the Delta group, but there was no significant difference in the LOS or disease duration. This finding suggested that vaccination may shorten the disease duration and LOS. In contrast to our findings, plasma levels of proinflammatory cytokines, including IL2, IL4 and TNFα, have been shown to increase in patients infected with the Wuhan variant [16]. We found that, among the three groups, there were no significant differences in any of the cytokines. According to previous studies, the NLR and PLR can also be used as significant biomarkers for predicting the prognosis of COVID-19 patients [21, 22]. Our study showed that the NLR and PLR were negatively correlated with disease duration and LOS between the Delta and Omicron groups in both patients and pediatric patients.

For the first time, we identified a chronic course of SARS-CoV-2 infection. Three patients coinfected with *Mtb* remained hospitalized for more than four months. Traditionally, infection and clearance of the virus is an acute process, usually occurring in approximately seven days, and even in patients with COVID-19, the hospital stay is only approximately 14 days, which is a much shorter period than that of Delta variant infections [23]. The presence of respiratory disease in patients leads to respiratory and pulmonary dysfunction, making *Mtb* a risk factor for COVID-19 [24]. Moreover, the primary target organs for SARS-CoV-2 and *M. tuberculosis* infection are the lungs, which results in double infections and damage to the lungs and consequent chronic disease.

Our research has several limitations. First, our case series may have presented a milder outcome for this disease and did not include severe patients. Because of strict preventive and control measures for the spread of SARS-CoV-2, many asymptomatic infected patients were admitted early for treatment and were included in the current study. Second, as there were only eleven Delta-infected patients coinfected with HIV, a more scientific study may require a larger sample size for validation.

Given the large number of patients in this study and those coinfected with other infectious diseases, our findings provide valuable information for understanding the clinical features of Delta variant infection. Due to the atypical nature of the initial symptoms in patients infected with Delta variants, an epidemiologic history investigation should be initiated as soon as a case arises. In addition, close contacts should be isolated and tested for nucleic acids. The government should conduct public daily education campaigns to prevent exposure to the Delta variant and encourage people not to go to crowded places to prevent its large-scale spread.

## Conclusion

In summary, we found that SARS-CoV-2 patients coinfected with *Mtb* or HIV exhibited a longer disease duration and LOS, with a decrease in B-cell and CD4 + T-cell counts, suggesting that these cells are related to immune function. Changes in cytokine levels suggest that coinfection with *Mtb* or HIV does not result in dysregulation of the immune response. Importantly, we found that coinfection with *Mtb* and SARS-CoV-2 could lead to a chronic course of infection for more than four months.

### Electronic supplementary material

Below is the link to the electronic supplementary material.

Supplementary Material 1

## Data Availability

The data that support the findings of this study are available from the First Affiliated Hospital of Zhengzhou University but restrictions apply to the availability of these data, which were used under license for the current study, and so are not publicly available. Data are however available from the authors upon reasonable request and with permission of the corresponding author.
