# Supplementary material for: Immune Dysregulation in SARS-CoV-2 patients coinfected with Mycobacterium tuberculosis (Mtb) or HIV in China
Source: BMC Public Health. 2024 Feb 22;24:556. doi: 10.1186/s12889-024-17905-3 (PMC10882883; doi:10.1186/s12889-024-17905-3)
Supplement: Supplementary file 1 — Supplementary Material 1 [file 12889_2024_17905_MOESM1_ESM.docx]

| Supplementary Table 1. Demographic and baseline clinical characteristics of patients infected with Delta without vaccination. | | | | | | |
| --- | --- | --- | --- | --- | --- | --- |
|  | Delta group  (n=40) | *Mtb* group  (n=34) | HIV group  (n=11) | *P1*-value | *P2*-value | *P3-*value |
| Characteristics | | | | | | |
| Median (interquartile) age (years) | 47 (19-58) | 35.5 (23.75-58.25) | 47 (32-51) | 0.997 | 0.971 | 0.993 |
| Age groups (years) |  |  |  | 0.047 | 0.0002 | 0.031 |
| ≤18 | 10 (25%) | 2 (5.9%) | 1 (9%) | ·· | ·· | ·· |
| 19-40 | 9 (22.5%) | 16 (47.1%) | 2 (18%) | ·· | ·· | ·· |
| 41-65 | 14 (35%) | 18 (35%) | 8 (73%) | ·· | ·· | ·· |
| ≥66 | 7 (17.5%) | 7 (20.6%) | 0 | ·· | ·· | ·· |
| Male sex | 19 (47.5%) | 25 (73.5%) | 8 (73%) | 0.023 | 0.138 | 1.0 |
| Familial cluster | 15 (37.5%) | 5 (14.7%) | 0 | 0.028 | 0.041 | 0.425 |
| Coexisting conditions |  |  |  |  |  |  |
| Diabetes | 8 (20%) | 6 (17.6%) | 5 (45%) | 0.797 | 0.058 | 0.044 |
| Hypertension | 5 (12.5%) | 2 (5.9%) | 1 (9%) | 0.568 | 1.0 | 1.0 |
| Cardiovascular disease | 6 (15%) | 3 (8.8%) | 0 | 0.65 | 0.401 | 0.565 |
| Chronic obstructive pulmonary disease | 0 | 0 | 0 | ·· | ·· | ·· |
| Fever | 9 (22.5%) | 12 (35.3%) | 3 (27%) | 0.224 | 1.0 | 0.902 |
| Highest temperature, °C |  |  |  | 0.007 | 1.0 | 0.149 |
| <37.3 | 31 (77.5%) | 23 (67.7%) | 8 (73%) | ·· | ·· | ·· |
| 37.3-38 | 6 (15%) | 5 (14.7%) | 1 (9%) | ·· | ·· | ·· |
| >38 | 3 (7.5%) | 6 (17.6%) | 2(18%) | ·· | ·· | ·· |
| Cough | 10 (25%) | 10 (29.4%) | 3 (27%) | 0.67 | 1.0 | 1.0 |
| Expectoration | 7 (17.5%) | 3 (8.8%) | 1(9%) | 0.455 | 0.833 | 1.0 |
| Myalgia or fatigue | 4 (10%) | 12 (35.3%) | 4 (36%) | 0.008 | 0.097 | 1.0 |
| Nasal congestion | 1 (2.5%) | 7 (20.6%) | 0 | 0.034 | 1.0 | 0.246 |
| Loss of smell and taste | 0 | 5 (14.7%) | 2 (18%) | 0.041 | 0.043 | 1.0 |
| Headache | 1 (2.5%) | 2 (5.9%) | 0 | 0.886 | 1.0 | 1.0 |
| Severity score |  |  |  | 0.257 | 1.0 | 0.829 |
| 4 | 29 (72.5%) | 25 (73.5%） | 8 (73%) | ·· | ·· | ·· |
| 5 | 11 (27.5%) | 7 (20.6%) | 3 (27%) | ·· | ·· | ·· |
| 9 | 0 | 2 (5.9%) | 0 | ·· | ·· | ·· |
| Dyspnea | 0 | 0 | 0 | ·· | ·· | ·· |
| The incubation period (days) | 4 (2-8.5) | 2 (1-3) | 4 (2-6) | 0.006 | 0.473 | 0.188 |
| Time from illness onset to first hospital admission (days) | 1.0 (0-3.75) | 5 (4-5) | 3 (2-4) | <0.0001 | 0.725 | 0.024 |
| *P1*-value comparing the Delta group and *Mtb* group, *P2*-value comparing the Delta group and HIV group and *P3*-value comparing *Mtb* group and HIV group are from χ², or ANOVA. LOS, length of stay. | | | | | | |

| Supplementary Table 2. Laboratory findings of Delta-infected patients who had not been vaccinated at admission to the hospital. | | | | | | |
| --- | --- | --- | --- | --- | --- | --- |
|  | Delta group  (n=40) | *Mtb* group  (n=34) | HIV group  (n=11) | *P1*-value | *P2*-value | *P3-*value |
| White blood cell, × 10⁹/L | 4.86 (4.12-6.41) | 4.19 (3.23-5.44) | 3.57 (2.58-5.46) | 0.072 | 0.034 | 0.375 |
| <4 | 9 (22.5%) | 14 (41.2%) | 6 (55%) | 0.257 | 0.182 | 0.67 |
| 4-10 | 30 (75%) | 20 (58.8%) | 5 (45%) | ·· | ·· | ·· |
| >10 | 1 (2.5%) | 0 | 0 | ·· | ·· | ·· |
| Neutrophil, × 10⁹/L | 2.57 (1.99-3.26) | 2.39 (1.76-3.1) | 1.99 (1.31-2.79) | 0.467 | 0.130 | 0.315 |
| Lymphocyte, × 10⁹/L | 1.49 (1.15-2.22) | 1.29 (0.79-1.67) | 1.15 (0.78-1.67) | 0.035 | 0.160 | 1.0 |
| <1.0 | 8 (20%) | 9 (26.5%) | 5 (45%) | 0.510 | 0.185 | 0.419 |
| Eosinophil, × 10⁹/L | 0.07 (0.02-0.11) | 0.06 (0.02-0.16) | 0.07 (0.03-0.17) | 0.436 | 0.682 | 0.838 |
| Platelet, × 10⁹/L | 156.5 (106.5-200) | 147.5 (106.8-217.5) | 155 (91-165) | 0.777 | 0.805 | 0.665 |
| <100 | 8 (20%) | 7 (20.6%) | 3 (27%) | 0.95 | 0.916 | 0.963 |
| ≥100 | 32 (80%) | 27 (79.4%) | 7 (64%) | ·· | ·· | ·· |
| Haemoglobin, g/L | 125.5 (119.3-146) | 129 (105-141) | 128 (107-140) | 0.271 | 0.648 | 0.768 |
| Prothrombin time, s | 11.8 (11.4-12.4) | 11.8 (11.4-12.8) | 11.8 (11.3-11.8) | 0.970 | 0.420 | 0.442 |
| D-dimer, mg/L | 0.39 (0.21-0.78) | 0.31 (0.24-0.83) | 0.60 (0.23-1.15) | 0.539 | 0.515 | 0.998 |
| ≥1.0 | 6 (15%) | 6 (17.6%) | 3 (27%) | 0.758 | 0.618 | 0.795 |
| Alanine aminotransferase, U/L | 16 (11-28) | 17 (10-26.5) | 17 (12-54) | 0.458 | 0.534 | 0.912 |
| Aspartate aminotransferase, U/L | 26 (19-32) | 26.5 (17.8-42.5) | 20 (15-36) | 0.101 | 1.0 | 0.146 |
| Albumin, g/L | 42.1 (38.5-45.2) | 40.6 (34.6-43.9) | 42.4 (38.6-45.3) | 0.057 | 0.736 | 0.102 |
| Total Bilirubin, μmol/L | 8.85 (5.5-12.53) | 9.4 (6.25-11.23) | 8.6 (5.2-11.8) | 0.324 | 0.533 | 0.958 |
| Blood urea nitrogen, μmol/L | 4.61 (3.84-5.29) | 4.88 (4.18-6.57) | 5.30 (4.88-6.14) | 0.239 | 0.505 | 1.0 |
| Creatinine, μmol/L | 59.5 (40.5-79.5) | 58.5 (52-74.5) | 71 (49-80) | 0.319 | 0.565 | 0.998 |
| ≥133 | 1 (2.5%) | 3 (8.8%) | 2 (18%) | 0.495 | 0.114 | 0.759 |
| Creatinine kinase, U/L | 63.5 (45.2-104.8) | 30.3 (23.7-79.9) | 47.5 (25.6-120.6) | 0.101 | 0.775 | 0.275 |
| Lactate dehydrogenase, U/L | 220.5 (189-269.8) | 219.5 (181.8-269.5) | 213 (165-271) | 0.548 | 0.943 | 0.421 |
| ≥250 | 13 (32.5%) | 12 (35.3%) | 3 (27%) | 0.8 | 1.0 | 0.902 |
| C-reactive protein, mg/L | 5.65 (1.18-22.7) | 9.1 (3.93-26.28) | 1.55 (0.85-2.53) | 0.599 | 0.515 | 0.319 |
| >10 | 14 (35%) | 17 (50%) | 0 | 0.192 | 0.485 | 0.132 |
| NLR | 1.67 (1.11-3.34) | 1.9 (1.41-3.43) | 1.49 (1.08-2.55) | 0.980 | 0.464 | 0.462 |
| PLR | 82.54 (66.47-125.78) | 116.96 (82.69-208.38) | 139.53 (62.60-178.41) | 0.962 | 0.851 | 0.829 |
| Pneumonia | 20 (50%) | 25 (73.5%) | 11 (100%) | 0.089 | 0.002 | 0.14 |
| Bilateral involvement of chest radiographs | 17 (42.5%) | 22 (64.7%) | 7 (64%) | 0.112 | 0.371 | 1.0 |
| *P1*-value comparing the delta group and TB group, *P2*-value comparing the delta group and HIV group and *P3*-value comparing *Mtb* group and HIV group are from χ², or ANOVA. NLR, neutrophil-lymphocyte ratio; PLR, planet-lymphocyte ratio. | | | | | | |

| Supplementary Table 3. Lymphocyte subpopulations and cytokines of Delta-infected patients without vaccination. | | | | | | |
| --- | --- | --- | --- | --- | --- | --- |
|  | Delta group  (n=40) | *Mtb* group  (n=34) | HIV group  (n=11) | *P1*-value | *P2-*value | *P3-*value |
| Lymphocyte subpopulations | | | | | | |
| Total T cells, per μl | 1057.0 (566.8-1429.5) | 719.0 (481.5-911.5) | 947 (484-1320) | 0.027 | 0.494 | 0.340 |
| Total T cells, % | 71.22 (63.14-74.98) | 70.99 (59.57-75.08) | 78.61 (74.10-80.38) | 0.244 | 0.466 | 0.890 |
| CD4^+^ T cells, per μl | 640.5 (428.5-830.0) | 411.0 (239.5-522.3) | 246 (56-540) | <0.0001 | 0.0008 | 0.375 |
| CD4^+^ T cells, % | 43.17 (35-76.64) | 40.68 (31.99-45.43) | 21.04 (4.28-36.26) | 0.235 | 0.154 | 0.603 |
| CD8^+^ T cells, per μl | 388.5 (223.5-483.3) | 235.5 (140.0-346.0) | 595 (340-973) | 0.051 | 0.007 | 0.0001 |
| CD8^+^ T cells, % | 25.65 (21.75-29.39) | 24.39 (19.25-30.71) | 52.68 (42.66-60.48) | 0.230 | 0.950 | 0.307 |
| CD4/CD8 ratio | 1.76 (1.41-2.12) | 1.48 (1.33-2.02) | 0.37 (0.07-0.94) | 0.198 | 0.097 | 0.498 |
| B cells, per μl | 182.0 (104.3-308.5) | 104.0 (78.0-174.8) | 136 (98-153) | 0.020 | 0.012 | 0.996 |
| B cells, % | 12.72 (10.98-18.17) | 10.98 (7.34-16.73) | 10.78 (7.57-16.16) | 0.201 | 0.134 | 0.574 |
| NK cells, per μl | 158.5 (90.5-257.5） | 137.0 (118.8-203.8) | 108 (64-180) | 0.287 | 0.121 | 0.463 |
| NK cells, % | 10.17 (7.75-18.31) | 12.49 (11.00-19.11) | 9.22 (7.08-11.34) | 0.484 | 0.306 | 0.028 |
| Cytokines | | | | | |  |
| IL-2 | 9.25 (6.85-18.65) | 10.45 (6.83-14.63) | 2.5 (2.5-8.65) | 0.575 | 0.264 | 0.490 |
| IL-4 | 6.25 (4.90-12.0) | 7.55 (6.38-11.2) | 2.5 (2.5-6.43) | 0.540 | 0.215 | 0.002 |
| IL-6 | 46.45 (42.28-61.88) | 51.10 (44.18-66.28) | 49.3 (45.38-64) | 0.366 | 0.509 | 0.996 |
| IL-10 | 5.1 (3.6-7.8) | 6.75 (5.20-10.93) | 2.5 (2.5-3.78) | 0.249 | 0.104 | 0.019 |
| IL-12p70 | 0.8 (0-1.95) | 1.2 (0.5-1.9) | 2.5 (1.1-2.5) | 0.434 | 0.511 | 0.105 |
| IL-17A | 147.7 (147.2-148.7) | 148.6 (147.5-151.3) | 148.1 (147.3-148.5) | 0.370 | 0.612 | 0.890 |
| IFN-γ | 46.4 (45.6-47.8) | 46.8 (46.0-49.2) | 102.4 (48-121.9) | 0.447 | 0.309 | 0.132 |
| TNF-a | 2.05 (0.93-2.58) | 2.5 (1.18-5.48) | 2.5 (1.7-2.5) | 0.345 | 0.824 | 0.201 |
| *P1*-value comparing the delta group and *Mtb* group, *P2*-value comparing the delta group and HIV group and *P3*-value comparing *Mtb* group and HIV group are from ANOVA. | | | | | | |

| Supplementary Table 4. Treatment and outcomes of patients infected with Delta without vaccination. | | | | | | |
| --- | --- | --- | --- | --- | --- | --- |
|  | Delta group  (n=40) | *Mtb* group  (n=34) | HIV group  (n=11) | *P1*-value | *P2-*value | *P3-*value |
| Admission to intensive care unit | 5 (12.5%) | 6 (17.6%) | 2 (18%) | 0.535 | 1.0 | 1.0 |
| Secondary infection | 0 | 1 (2.9%) | 0 | 0.459 | ·· | 1.0 |
| Shock | 0 | 2 (5.9%) | 0 | 0.208 | ·· | 1.0 |
| Treatment | | | | | | |
| TCM treatment | 34 (85%) | 31 (91.2%) | 11 (100%) | 0.65 | 0.401 | 0.565 |
| Immunotherapy | 15 (37.5%) | 25 (73.5%) | 9 (82%) | 0.002 | 0.009 | 0.879 |
| Antiviral treatment | 4 (10%) | 21 (61.8%) | 5 (45%) | <0.0001 | 0.022 | 0.548 |
| Antibiotics | 8 (20%) | 27 (79.4%) | 2 (18%) | <0.0001 | 1.0 | 0.001 |
| Heparin | 11 (27.5%) | 3 (8.8%) | 6 (55%) | 0.041 | 0.062 | 0.01 |
| Plasma of recovered patients | 1 (2.5%) | 2 (5.9%) | 1 (9%) | 0.886 | 0.388 | 1.0 |
| Corticosteroid and gamma globulin | 0 | 2 (5.9%) | 0 | 0.208 | 0.216 | 1.0 |
| Glucocorticoids alone | 0 | 6 (17.6%) | 0 | 0.019 | ·· | 0.324 |
| Continuous renal-replacement therapy | 0 | 3 (8.8%) | 0 | 0.185 | ·· | 0.565 |
| Oxygen support | | | | | | |
| Nasal cannula | 11 (27.5%) | 5 (14.7%) | 2 (18%) | 0.183 | ·· | 1.0 |
| Non-invasive ventilation or high-flow nasal cannula | 0 | 4 (11.8%) | 1 (9%) | 0.086 | 0.216 | 1.0 |
| Invasive mechanical ventilation | 0 | 2 (5.9%) | 0 | 0.208 | 1.0 | 1.0 |
| Invasive mechanical ventilation and ECMO | 0 | 2 (5.9%) | 0 | 0.208 | 1.0 | 1.0 |
| Prognosis | | | | | | |
| Hospitalisation | 0 | 0 | 0 | ·· | ·· | ·· |
| Discharge | 40 (100%) | 34 (100%) | 11 (100%) | ·· | ·· | ·· |
| Death | 0 | 0 | 0 | ·· | ·· | ·· |
| LOS, days | 29 (20.3-48.3) | 45 (20.3-87.8) | 51(40-71) | 0.104 | 0.165 | 0.806 |
| Disease duration | 30.5 (22.5-50) | 49.5 (27.8-92.3) | 55(40-73) | 0.055 | 0.150 | 0.927 |
| TCM treatment, traditional Chinese medicine treatment; LOS, length of stay. | | | | | | |

| Supplementary Table 5. Demographic and baseline clinical characteristics of pediatric patients in the Delta and Omicron groups. | | | |
| --- | --- | --- | --- |
|  | Delta group (n=11) | Omicron group (n=235) | *P*-value |
| Patients under 18 years | | | |
| Characteristics |  |  |  |
| Median (interquartile) age (years) | 7.60 ± 5.38 | 15.91 ± 3.25 | <0.0001 |
| Male sex | 6 (55%) | 113 (48%) | 0.675 |
| Familial cluster | 5 (45%) | 87 (37%) | 0.912 |
| Underlying disease |  |  |  |
| Any | 0 | 0 | ·· |
| Number of vaccination doses |  |  | ·· |
| 0 | 7 (100%) | 4 (100%) | ·· |
| 1 | 0 | 0 | ·· |
| 2 | 0 | 0 | ·· |
| 3 | 0 | 0 | ·· |
| Signs and symptoms |  |  |  |
| Fever | 2 (18%) | 53 (22.6%) | 1.0 |
| Highest temperature, °C |  |  | 0.41 |
| <37.3 | 9 (82%) | 182(77.5%) | ·· |
| 37.3-38 | 1 (9%) | 24 (10.2%) | ·· |
| >38 | 1 (9%) | 29 (12.3%) | ·· |
| Cough | 1 (9%) | 144 (63%) | 0.001 |
| Expectoration | 1 (9%) | 68 (29.6%) | 0.276 |
| Myalgia or fatigue | 0 | 8 (3%) | 1.0 |
| Nasal congestion | 1 (9%) | 70 (29.8%) | 0.254 |
| Loss of smell and taste | 0 | 3 (1.3%) | 1.0 |
| Headache | 1 (9%) | 4 (1.7%) | 0.206 |
| Dyspnea | 0 | 3 (1.3%) | 1.0 |
| Incubation period (days) | 6.30 ± 4.62 | 0.48 ± 1.32 | <0.0001 |
| Days from illness onset to first hospital admission | 1.55 ± 1.97 | 2.44 ± 1.90 | 0.059 |
| Systolic pressure, mmHg | 108.8 ± 13.60 | 115.3± 11.08 | 0.183 |
| Pneumonia | 0 | 41 (17.4%) | 0.27 |
| Bilateral involvement of chest radiographs | 0 | 13 (5.5%) | 1.0 |
| Data are median (IQR), mean ± SEM or n (%). *P*-values comparing non-chronic and chronic groups are from χ² test, Fisher’s exact test, or Mann-Whitney U test. TB, Tuberculosis. | | | |

| Supplementary Table 6. Differences in laboratory findings between the Delta and Omicron groups of pediatric patients. | | | |
| --- | --- | --- | --- |
|  | Delta group (n=11) | Omicron group (n=235) | *P*-value |
| Patients under 18 years | | | |
| White blood cell, × 10⁹/L | 6.83 ± 3.35 | 5.29 (4.40-6.34) | 0.078 |
| <4 | 0 | 41 (17.4%) | 0.27 |
| 4-10 | 10 (91%) | 191 (81.3%) | ·· |
| >10 | 1 (9%) | 3 (1.3%) | ·· |
| Neutrophil, × 10⁹ /L | 2.80 ± 2.91 | 2.47 (1.80-3.32) | 0.134 |
| Lymphocyte, × 10⁹ /L | 3.41 ± 1.32 | 2.04 (1.61-2.50) | 0.0003 |
| <1.0 | 0 | 5 (2.1%) | 1.0 |
| Eosinophil, × 10⁹ /L | 0.12 ± 0.07 | 0.11 (0.06-0.19) | 0.862 |
| Platelet, × 10⁹/ L | 202.9 ± 66.1 | 211 (175-245) | 0.765 |
| <100 | 1 (9%) | 0 | 0.045 |
| ≥100 | 10 (91%) | 235 (100%) | ·· |
| Haemoglobin, g/L | 149.3 ± 33.5 | 146 (132-162) | 0.001 |
| Prothrombin time, s | 12.06 ± 0.60 | 12.0 (11.3-12.8) | 0.836 |
| D-dimer, mg/L | 4.02 ± 10.87 | 0.04 (0.01-0.09) | <0.0001 |
| ≥1.0 | 2 (18%) | 2 (0.9%) | 0.01 |
| Alanine aminotransferase, U/L | 20.06 ± 15.96 | 15 (10-22.25) | 0.16 |
| Aspartate aminotransferase, U/L | 26.82 ± 6.01 | 18 (15-22) | 0.0005 |
| Albumin, g/L | 46.01 ± 3.59 | 43 (41.98-45.8) | 0.094 |
| Total Bilirubin, μmol/L | 11.69 ± 4.97 | 10.95 (8.3-14.3) | <0.0001 |
| Blood urea nitrogen, μmol/L | 4.53 ± 0.86 | 4.3 (3.6-5.1) | 0.494 |
| Creatinine, μmol/L | 37.45 ± 22.35 | 70 (63-80) | <0.0001 |
| ≥133 | 0 | 0 | ·· |
| Creatinine kinase, U/L | 73.91 ± 48.77 | 91 (69-119) | 0.002 |
| Lactate dehydrogenase, U/L | 246.36 ± 65.46 | 159 (139.5-182.5) | <0.0001 |
| ≥250 | 5 (45%) | 6 (2.6%) | <0.0001 |
| C-reactive protein, mg/L | 15.86 ± 39.58 | 1.21 (0.67-3.55) | 0.859 |
| >10 | 1 (9%) | 9 (3.8%) | 0.373 |
| NLR | 0.95 ± 0.97 | 1.18 (0.81-1.65) | 0.007 |
| PLR | 65.50 ± 29.56 | 98.92 (80.59-132) | 0.0002 |

| Supplementary Table 7. Treatments and outcomes of pediatric patients in the Delta and Omicron groups. | | | |
| --- | --- | --- | --- |
|  | Delta group (n=11) | Omicron group (n=235) | *P*-value |
| Admission to the intensive care unit | 1 (9%) | 0 | 0.045 |
| Secondary infection | 0 | 0 | ·· |
| Shock | 0 | 0 | ·· |
| Treatment | | | |
| TCM treatment | 6 (55%) | 234 (99.6%) | <0.0001 |
| Immunotherapy | 2 (18%) | 97 (41.3%) | 0.225 |
| Antiviral treatment | 1 (9%) | 0 | 0.045 |
| Antibiotics | 1 (9%) | 6 (2.6%) | 0.277 |
| Heparin | 0 | 14 (6.0%) | 0.641 |
| Plasma of recovered patients | 0 | 0 | ·· |
| Corticosteroid and gamma globulin | 0 | 0 | ·· |
| Glucocorticoids alone | 0 | 0 | ·· |
| Continuous renal-replacement therapy | 0 | 0 | ·· |
| Oxygen support | | | |
| Nasal cannula | 0 | 0 | ·· |
| Non-invasive ventilation or high-flow nasal cannula | 0 | 0 | ·· |
| Invasive mechanical ventilation | 0 | 0 | ·· |
| Invasive mechanical ventilation and ECMO | 0 | 0 | ·· |
| Prognosis | | | |
| Hospitalisation | 0 | 0 | ·· |
| Discharge | 40 (100%) | 34 (100%) | ·· |
| Death | 0 | 0 | ·· |
| LOS, days | 28.8 ± 11.9 | 20 (15-25) | 0014 |
| Disease duration | 30.4 ± 11.8 | 22 (17-28) | 0.044 |
| ECMO=extracorporeal membrane oxygenation. TCM treatment=traditional Chinese medicine treatment. TB, Tuberculosis. | | | |

| Supplementary Table 8. Demographic and baseline clinical characteristics of *Mtb* patients with Delta according to disease duration. | | | |
| --- | --- | --- | --- |
|  | Non-chronic patients (n=49) | Chronic patients (n=3) | *P*-value |
| Characteristics |  |  |  |
| Median (interquartile) age (years) | 48.0 (26.0-63.5) | 42.3 ± 14.2 | 0.78 |
| Age groups (years) |  |  | 1.0 |
| ≤18 | 2 (4%) | 0 | ·· |
| 19-40 | 20 (41%) | 1 (33%) | ·· |
| 41-65 | 17 (35%) | 2 (67%) | ·· |
| ≥66 | 10 (20%) | 0 | ·· |
| Male sex | 35 (71%) | 2 (67%) | 1.0 |
| Familial cluster | 7 (14%) | 1 (33%) | 0.40 |
| Underlying disease |  |  |  |
| Any | 49 (100%) | 3 (100%) | ·· |
| Diabetes | 10 (20%) | 0 | 1.0 |
| Hypertension | 3 (6%) | 0 | 1.0 |
| Cardiovascular disease | 3 (6%) | 0 | 1.0 |
| Chronic obstructive pulmonary disease | 1 (2%) | 0 | 1.0 |
| Number of vaccination doses |  |  | 1.0 |
| 0 | 32 (65%) | 2 (67%) | ·· |
| 1 | 15 (31%) | 1 (33%) | ·· |
| 2 | 2 (4%) | 0 | ·· |
| 3 | 0 | 0 | ·· |
| Signs and symptoms |  |  |  |
| Fever | 12 (24%) | 1 (33%) | 1.0 |
| Highest temperature, °C | 36.6 (36.5-37.2) | 37.4 ± 1.4 | 0.50 |
| <37.3 | 38 (78%) | 2 (67%) | 0.15 |
| 37.3-38 | 6 (12%) | 0 |  |
| >38 | 5 (10%) | 1 (33%) |  |
| Cough | 13 (27%) | 1 (33%) | 1.0 |
| Expectoration | 5 (10%) | 0 | 1.0 |
| Myalgia or fatigue | 19 (39%) | 0 | 0.46 |
| Nasal congestion | 9 (18%) | 0 | 1.0 |
| Loss of smell and taste | 4 (8%) | 1 (33%) | 0.27 |
| Headache | 3 (6%) | 0 | 1.0 |
| Dyspnea | 0 | 0 |  |
| Incubation period (days) | 2.0 (1.0-3.0) | 2.3 ± 1.5 | 0.82 |
| Days from illness onset to first hospital admission | 5.0 (4.0-5.0) | 4.7 ± 1.5 | 0.65 |
| Systolic pressure, mmHg | 120.0 (113.0-124.5) | 122 ± 5 | 0.43 |
| Pneumonia | 36 (73%) | 3 (100%) | 0.56 |
| Bilateral involvement of chest radiographs | 29 (59%) | 3 (100%) | 0.42 |
| Data are median (IQR), mean ± SEM or n (%). *P*-values comparing non-chronic and chronic groups are from χ² test, Fisher’s exact test, or Mann-Whitney U test. TB, Tuberculosis. | | | |

| Supplementary Table 9. Laboratory findings of *Mtb* patients with Delta according to disease duration. | | | |
| --- | --- | --- | --- |
|  | Non-chronic patients (n=49) | Chronic patients (n=3) | *P*-value |
| White blood cell, × 10⁹/L | 4.07 (3.22-5.40) | 4.14 ± 3.56 | 0.68 |
| <4 | 23 (47%) | 2 (67%) | 0.95 |
| 4-10 | 25 (51%) | 1 (33%) | ·· |
| >10 | 1 (2%) | 0 | ·· |
| Neutrophil, × 10⁹ /L | 2.45 (1.65-3.11) | 3.15 ± 3.03 | 0.86 |
| Lymphocyte, × 10⁹ /L | 1.31 (0.92-1.60) | 0.59 ± 0.34 | 0.054 |
| <1.0 | 14 (29%) | 3 (100%) | 0.23 |
| Lymphocyte percentage, % | 31.3 (20.25-40.20) | 18.23 ± 2.33 | 0.10 |
| <20 | 10 (20%) | 1 (33%) | 0.52 |
| Eosinophil, × 10⁹ /L | 0.07 (0.025-0.16) | 0.026 ± 0.03 | 0.106 |
| Platelet, × 10⁹/ L | 145 (108-200) | 125.3 ± 48.3 | 0.53 |
| <100 | 8 (16%) | 1 (33%) | 0.44 |
| ≥100 | 41 (84%) | 2 (67%) |  |
| Prothrombin time (s) | 11.8 (11.5-12.85) | 11.3 ± 0.4 | 0.091 |
| D-dimer (mg/L) | 0.31 (0.25-0.81) | 0.39 ± 0.40 | 0.46 |
| ≥1.0 | 9 (18%) | 0 | 1.0 |
| Albumin (g/L) | 40.6 (35.65-44.15) | 37.1 ± 2.27 | 0.22 |
| <30 | 3 (6%) | 0 | 1.0 |
| Aspartate aminotransferase (U/L) | 26.0 (19.0-44.0) | 47.3 ± 36.1 | 0.44 |
| >40 | 13 (27%) | 1 (33%) | 1.0 |
| Direct Bilirubin (μmol/L) | 2.70 (2.05-4.50) | 3.06 ± 0.95 | 1.0 |
| Creatinine (μmol/L) | 61.0 (52.5-73.5) | 44.7 ± 22.7 | 0.18 |
| ≥133 | 3 (6%) | 0 | 1.0 |
| Creatinine kinase (U/L) | 34.3 (23.7-75.3) | NA |  |
| Lactate dehydrogenase (U/L) | 219.0 (175.0-266.0) | 314 ± 136.7 | 0.19 |
| ≥250 | 15 (31%) | 2 (67%) | 0.51 |
| Blood urea nitrogen (μmol/L) | 4.97 (4.23-6.43) | 4.16 ± 1.64 | 0.20 |
| C-reactive protein (mg/L) | 8.6 (3.1-26.7) | 51.0 ± 64.9 | 0.37 |
| >10 | 22 (45%) | 2 (67%) | 0.89 |
| NLR | 1.79 (1.32-3.37) | 5.12 ± 3.67 | 0.071 |
| PLR | 114.89 (78.05-189.37) | 108.8 ± 255.5 | 0.047 |

| Supplementary Table 10. Treatments and outcomes of *Mtb* patients with Delta according to disease duration. | | | |
| --- | --- | --- | --- |
|  | Non-chronic patients (n=49) | Chronic patients (n=3) | *P*-value |
| Admission to the intensive care unit | 7 (14%) | 1 (33%) | 0.40 |
| Secondary infection | 1 (2%) | 0 | 1.0 |
| Shock | 2 (4%) | 0 | 1.0 |
| Oxygen support |  |  |  |
| Nasal cannula | 7 (14%) | 0 | 1.0 |
| Non-invasive ventilation or high-flow nasal cannula | 4 (8%) | 1 (33%) | 0.27 |
| Invasive mechanical ventilation | 2 (4%) | 0 | 1.0 |
| Invasive mechanical ventilation and ECMO | 2 (4%) | 0 | 1.0 |
| ECMO=extracorporeal membrane oxygenation. TCM treatment=traditional Chinese medicine treatment. TB, Tuberculosis. | | | |
